# Network diffusion model reveals recovery multipliers and heterogeneous spatial effects in post-disaster community recovery

**DOI:** 10.1038/s41598-023-46096-x

**Published:** 2023-11-03

**Authors:** Chia-Fu Liu, Ali Mostafavi

**Affiliations:** Zachry Department of Civil and Environmental Engineering, Texas A&M University, 199 Spence St., College Station, TX 77843-3136 USA

**Keywords:** Climate-change impacts, Climate-change mitigation

## Abstract

Community recovery from hazards occurs through various diffusion processes within social and spatial networks of communities. Existing knowledge regarding the diffusion of recovery in community socio-spatial networks, however, is rather limited. To bridge this gap, we created a network diffusion model to characterize the unfolding of population activity recovery in spatial networks of communities. In particular, this study aims to answer the research question “To what extent can the diffusion model capture the spatial patterns of recovery?” Using population activity recovery data derived from location-based information associated with 2017 Hurricane Harvey in the Houston area, we parameterized the threshold-based network diffusion model using the genetic algorithm and then simulated the recovery diffusion process. The results show that the spatial effects of recovery are rather heterogeneous across different areas; some spatial areas demonstrate a greater spatial effect in their recovery. Also, the results show that low-income and minority areas are community recovery multipliers; with faster recovery in these areas corresponding to accelerated recovery for the entire community. Hence, prioritizing these areas in resource allocation during recovery has the potential to accelerate could expedite the recovery of the entire community’s recovery process while promoting recovery equality and equity.

## Introduction

Recovery from a disaster is a dynamic and multifaceted process that is critical to community resilience in the face of hazards and crises^1–3^. The pace of disaster recovery can vary significantly from location to location, even if those locations are affected by the same event, recovery^4^. Unfortunately, many recovery studies lack the necessary spatial and temporal granularity to fully understand the local variations in the recovery process and patterns. While previous research has examined factors that influence variability in recovery, such as the role of social capital^5–7^ and socio-demographic attributes^8–10^ of a community after disasters, there remains a lack of methodologies or metrics to capture trends of recovery across the entire affected area and over longer periods of time^11^.

This study examines the influence of socio-spatial network processes on post-disaster recovery, a topic that has received increasing attention, particularly in the context of disaster resilience. For example, one study^12^ examined social connectedness embedded in the community spatial network to characterize an area’s resourcefulness during a disaster. The proposed metric, hazard exposure heterophily, has the potential to influence community resilience. Another study^13^ examined the community characteristics within human mobility networks during a managed power outage resulting from the extreme weather event. The analysis results found that population movements were influenced by socio-demographic homophily, heterophilic hazard exposure, and the strength of social connectedness. This study highlights the importance of considering latent characteristics within socio-spatial networks for understanding managed power outage patterns. These results demonstrate the significance of socio-spatial networks as structures upon which various community resilience processes unfold. Of significance for community resilience is the recovery process. In fact, community recovery unfolds upon the socio-spatial networks embedded in communities^14^. However, existing knowledge lacks a comprehensive understanding of how recovery diffuses within community spatial networks. In this study, we define the diffusion of recovery as the spatial progression of recovery among different areas. Specifying the existence and understanding of the characteristics of network diffusion in post-disaster recovery can reveal insights regarding spatial effects and properties that could improve and expedite recovery. It is this knowledge gap that motivates the research questions addressed in this study.

To bridge this gap, our study uses a network diffusion model to examine the extent of spatial contiguity effects in the spread of recovery across different spatial areas within a community. Our basic premise, which underlines that the process of population activity recovery unfolds upon the spatial area, is grounded in Space Syntax theory^15^. This theory assumes that the spatial configuration of cities, often referred to as the urban grid, acts as an enabler for many of the socioeconomic interactions that define urban life. We measure the empirical recovery duration of spatial areas based on the population activity recovery^16^, using the time for population visitations to points of interest (POI) to return to the pre-disaster patterns as a proxy for recovery. In the recovery phase, the reopening of local POIs serves as an incentive and stimulates population activity in the neighborhood area. This phenomenon can be conceptualized as a form of recovery diffusion, where the resurgence of these local POIs contributes to the broader process of community recovery.

In the selection of the network diffusion model, our work is inspired by the broader field of network contagion and diffusion modeling. Network diffusion has been studied in the context of the processes for spreading knowledge^17^, virus transmission^18,19^, ideas^20^, and new behaviors^21^ across social, natural, and physical networks. The study of diffusion phenomena has paved the way for a better understanding of the dynamics of complex networks in a variety of scientific fields. Various theoretical network diffusion models have been proposed. For instance, in the SIR model^22^, which is a classic model of epidemic spread, network diffusion is modeled based on various states a node can have throughout the duration of an epidemic: susceptible (S), infected (I), and recovered (R) states. Other epidemic spread models proposed by Kermack include the SI and SIS models^22^. The SI (susceptible–infected) model assumes that once a node becomes infected, it remains infected, while in the SIS (susceptible–infected–susceptible) model, an infected person has a probability of becoming susceptible again. Network diffusion models have been adopted in examining other network spread processes. An example is the spread of the digital virus, where the malicious software tries to transfer a copy of itself from one mobile device to another^18,19^. Another example is the social contagion, which illustrates the spread of knowledge and innovation in a social network where people are deciding to adopt new concepts or innovations^20^.

In addition to the SIR models and their variations, the other two main types of diffusion models include the threshold model^23^ and the Independent Cascade model^24^. In this study, we use the threshold diffusion model in examining the spatial diffusion of recovery, which enables us to examine spatial contiguity effects. In the threshold model (first introduced by Granovetter^23^), a node has two mutually exclusive states (binary states); it may elect to adopt a certain behavior, such as whether to take part in a riot. The node’s state depends on the percentage of its neighbors (i.e., the threshold value) which have the same state. The model works as follows: each node starts with its own threshold and state (e.g., recovered and not recovered). During generation *t*, each node is observed: if the percentage of its neighbors recovered at time *t* – 1 exceeds its threshold, it will also become recovered. Based on this model, we can estimate the threshold values for each spatial area based on the empirical recovery data and evaluate the extent of spatial contiguity effects across different spatial areas.

In the first optimization step, the network diffusion model is parameterized by the genetic algorithm (GA) using empirical data from Hurricane Harvey in 2017 in the greater Houston area (Harris County, Texas). We use this model to evaluate the extent of spatial contiguity effects (i.e., spatial effects in the diffusion of recovery) and the extent to which spatial effects are homogeneous across the community. In the second optimization step, the GA is used to identify recovery multipliers based on the parameterized model derived in the previous step. These multipliers identify spatial areas whose recovery would expedite the recovery of the entire community. Understanding the role of recovery multipliers can inform resource allocation strategies and prioritize spatial areas to expedite overall community recovery. The following section describes the details of the community recovery data and the threshold diffusion model of community recovery.

## Materials and methods

### Population activity recovery data

In this study, we estimate the recovery of spatial areas based on population activity patterns. All methods were carried out in accordance with relevant guidelines and regulations. Prior studies^16,25^ have shown that examining the fluctuations in population visitations to POI can provide important insights about the extent of impact and the duration of recovery of population activities. While population activity recovery may not capture all aspects of population post-disaster recovery, it can serve as a proxy indicating an important recovery milestone. Population activity fluctuations capture the combined effects of disruptions in households’ lifestyles, infrastructure services, and businesses. Hence, the return of population activities to the pre-disaster steady state could be a reliable measure of aspects of community recovery. Also, through the use of location intelligence data, population activity recovery duration can be determined at fine spatial scales, such as at the census block group level. Such measurement of recovery duration at such a fine spatial scale is barely possible using standard methods of community recovery measurements based on surveys.

To specify the duration of population activity recovery, we followed the same data processing and method proposed in the study^16^. We analyzed the de-identified and aggregated location data to characterize the pattern of population activity recovery in Harris County, Texas (USA) following Hurricane Harvey in 2017. Hurricane Harvey made landfall in August 2017, severely impacting the Texas coastal area. The extensive flooding rendered many essential and non-essential facilities inaccessible, significantly impacting post-disaster activities. Therefore, this scenario provides an appropriate context for studying the pattern of population recovery.

We first analyzed the human mobility data obtained from Spectus^22^, a commercial provider of aggregated and anonymized mobility data. The human mobility dataset was collected through the CCPA- and GDPR-compliant framework and presented as the daily number of visits to each census block group to various POIs to specify fluctuations in population activity patterns. Second, POIs were categorized into essential (such as gasoline stations, grocery, and pharmacies) and non-essential POIs (such as banks, restaurants, and recreational centers). Third, the baseline was set for each group of POIs to measure population activity change due to Harvey. The baseline was obtained by averaging the number of visits from August 1 through August 21, 2017. Finally, the study calculated a 7-day moving average for each day from August 27 to December 3, spanning 14 weeks. This moving average included 3 days before the target day and 3 days after the target day. The study defined a community as recovered when they observed post-disaster activities for 3 days at 90% of the baseline level. In this study, we use the population activity recovery time of CBGs in terms of visits to essential POIs to develop the network diffusion model. The analysis is based on aggregating the recovery duration of users in each CBG. CBGs under the minimum visit count of four are not included in the dataset, making 2010 out of 2144 CBGs in Harris County available for use in the implementation of the network diffusion model. In the next section, we will present the network diffusion model.

### Recovery network diffusion model configuration

**Figure 1:**
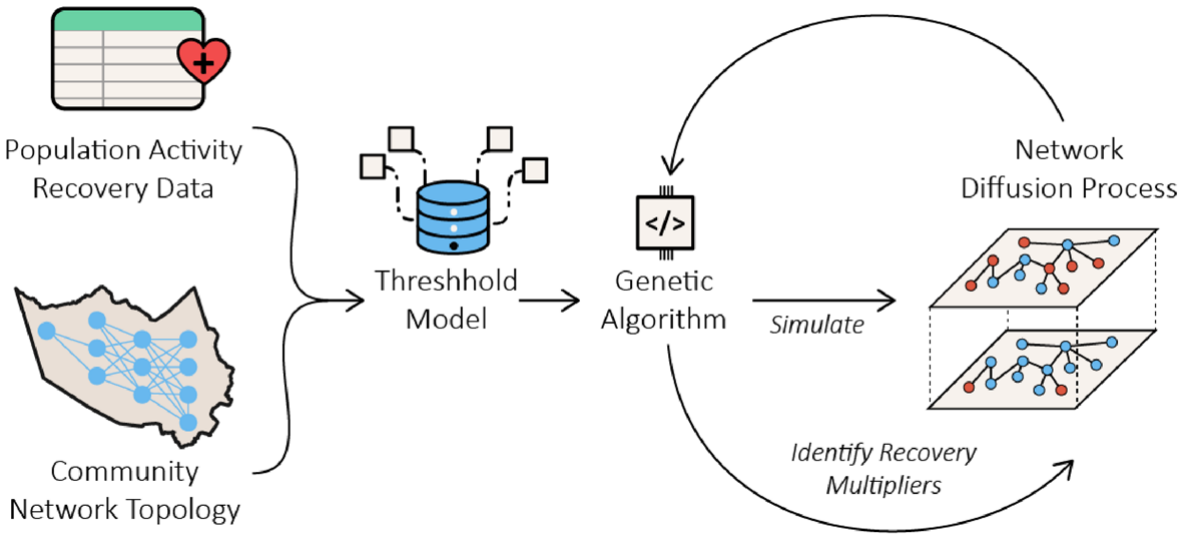
Overview of the steps for modeling post-disaster recovery as a network diffusion process.

**Figure 2:**
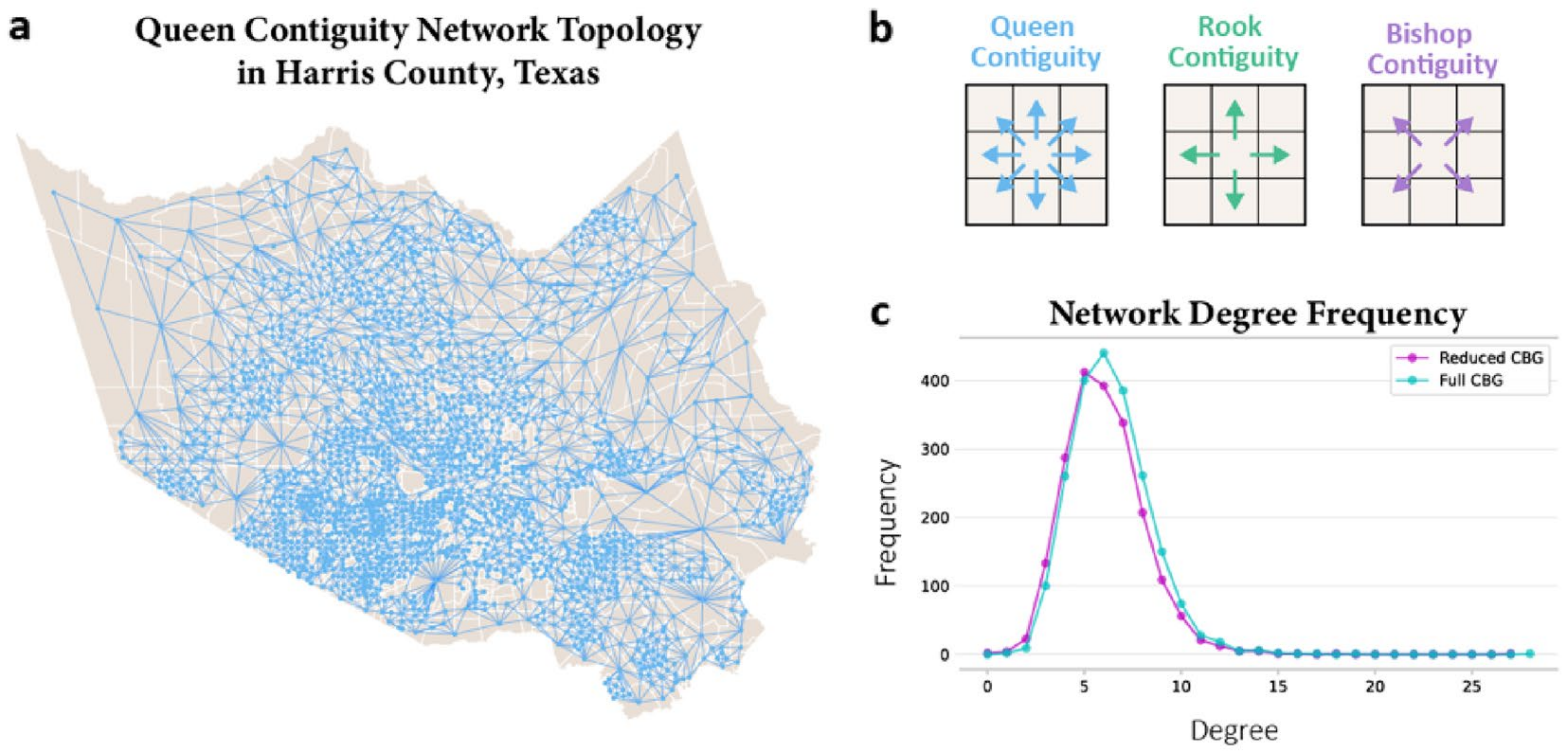
(**a**) Queen contiguity spatial network topology for the 2010 CBGs in Harris County, Texas. (**b**) The illustration of queen, rook, and bishop contiguity. (**c**) The node degree distribution for full CBG (2144) and reduced CBG (2010) in Harris County, Texas.

This section presents the steps in constructing and implementing the network diffusion model (Fig. 1). To develop the network diffusion model for population activity recovery, we first construct the spatial network topology $G=(\mathcal{V},\mathcal{E})$ for 2010 CBGs in Harris County, Texas. To characterize the spatial structure, several spatial proximity criteria for spatial data include queen, rook, and bishop contiguity. Rook contiguity defines neighbors based on the presence of a common edge, while bishop contiguity defines neighbors based on a common vertex between two spatial units. Queen contiguity, the most comprehensive of the three criteria, defines neighbors as spatial units that share a common edge or a common vertex. Since recovery diffusion can occur in multiple directions, the queen neighborhood structure was used in this study. Specifically, we used the queen neighborhood structure to construct the undirected graph by specifying the CBGs as vertices and the queen neighborhood as edges. As shown in Fig. 2, there are a total of 2010 vertices and 6079 edges in Harris County’s queen topological network, with an average node degree *k* of 6.049 and a graph density *d* of 0.00301.

In this study, we implemented NDlib (Network Diffusion Library)^26^ to model the population activity recovery as a network diffusion process. NDlib is a Python package built upon the NetworkX Python library and is a framework designed to describe, simulate, and study diffusion processes in complex networks. We use the threshold model^23,24^ to simulate the trajectory of the community recovery process in the spatial network. In a threshold model, a node has two distinct and mutually exclusive states, which are *affected* and *recovered*. Each node’s change in the state depends on the percentage of its neighbors that have the same state, which establishes a state change threshold. A set of initially recovered nodes and the threshold for each node are specified before the diffusion process begins. During a generation, each node is observed: if the percentage of its recovered neighbor is greater than its threshold, it will also be recovered.

**Figure 3:**
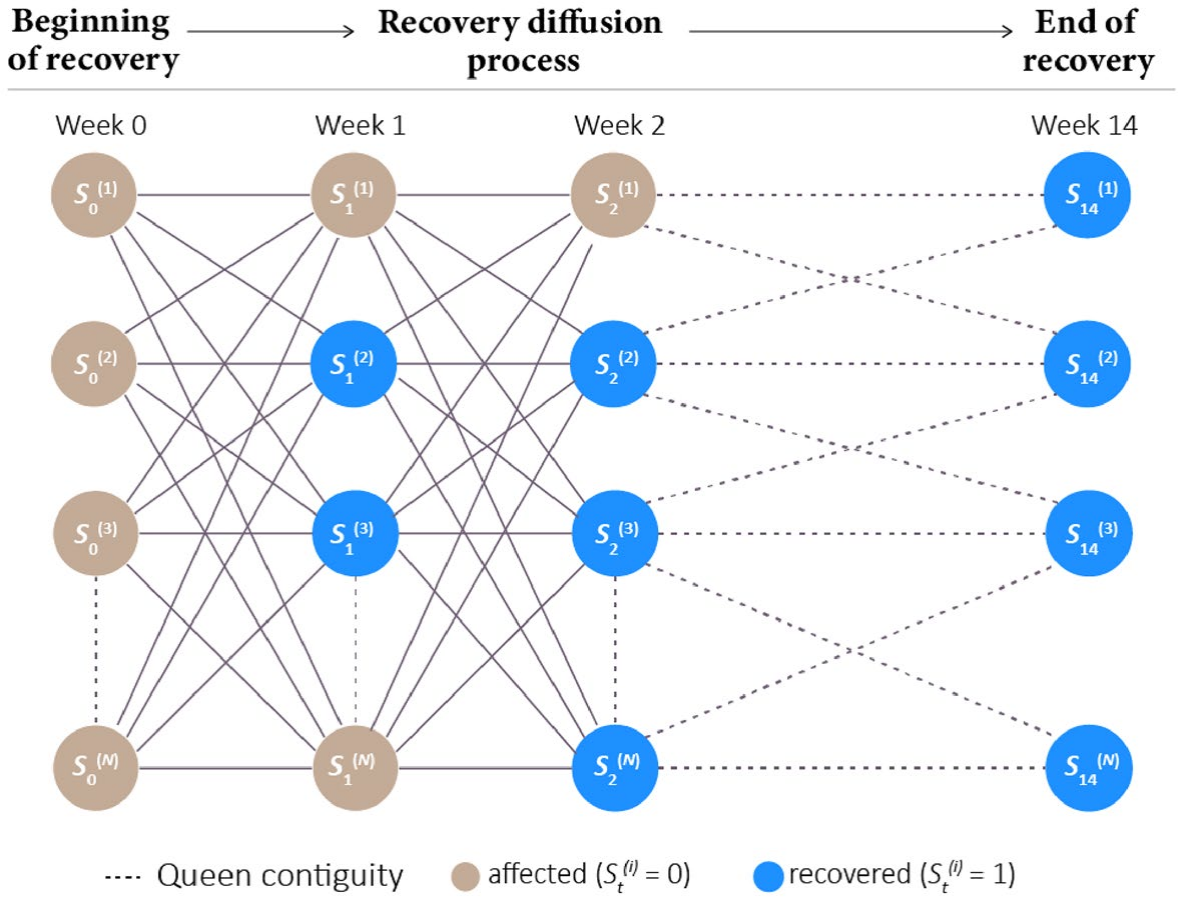
The recovery diffusion process implemented by the threshold model. A CBG ${\nu }_{i}$ would become recovered at week *t* + 1 if $\frac{\sum_{j\in \mathcal{N}({\nu }_{i})}{S}_{t}^{(j)}}{\#\mathcal{N}({\nu }_{i})}\ge {\tau }_{i}$, where $\mathcal{N}({\nu }_{i})$ is set of the neighbor of ${\nu }_{i}$.

The model implemented for the recovery process works as follows. Each CBG ${\nu }_{i}$, it has a threshold ${\tau }_{i}\in$ [0, 1] throughout the diffusion process along with the status ${S}_{t}^{(i)}$ $\in$ {0: *affected*, 1: *recovered*} at the end of week *t*. In particular, *t* $\in$ {0, 1, …, 14} and *t* = 0 signifies the end of the hazard event (Harvey rainfall stopped), which represents the beginning of the recovery phase. In our model, all the CBGs are defined as affected at *t* = 0. Since the minimum recovery duration after Hurricane Harvey was 1.14 weeks (population activities were affected in all areas regardless of flood impacts), we set the 750 CBGs whose recovery duration is less than 2 weeks to have threshold ${\tau }_{i}$ equal to zero, making them the first group of CBGs to recover at the end of week 2. The goal of the recovery diffusion model is to simulate the observed recovery duration of each CBG through the underlying network structure and estimate the threshold values for each CBG. The greater the threshold for a CBG, the higher the spatial effects in recovery of the CBG. The spatial effect captures the extent of spatial interdependence among the areas during recovery which is the extent to which an area relies on resources and facilities of neighboring areas for recovery. Figure 3 shows the progression of the recovery diffusion process.

To simulate the empirical recovery time of each CBG by parameterizing $\tau$, we formulate the objective function of the optimized diffusion model as follows:

1 $$\underset{\tau }{\mathrm{min}}L\left(S,\widehat{S}\right)=\sum_{t=1}^{14}\sum_{i=1}^{2010}I({S}_{t}^{(i)}\ne {\widehat{S}}_{t}^{(i)})$$

where ${S}_{t}^{(i)}$ is CBG ${\nu }_{i}$'s state at time *t* observed from the empirical data while ${\widehat{S}}_{t}^{(i)}$ is CBG ${\nu }_{i}$'s state at time *t* generated from the diffusion model. $L(\cdot )$ is defined as a 0–1 loss function and $I(\cdot )$ is the indicator function defined as

2 $$I\left(\cdot \right):=\left\{\begin{array}{ll}1 &\quad if\, {S}_{t}^{(i)}\ne {\widehat{S}}_{t}^{(i)}\\ 0 &\quad if\, {S}_{t}^{(i)}={\widehat{S}}_{t}^{(i)}\end{array}\right.$$

Mathematically, our goal is to obtain a set of threshold values $\tau$ which will generate minimal loss (based on observed recovery durations) throughout the entire diffusion process. The notations in the diffusion model and their descriptions are summarized in Table 1.

**Table 1:** List of major symbols and descriptions in the network diffusion model.

Sym	Domain	Description
$\mathcal{V}$	${\mathbb{N}}^{n}$	A set of vertices with size *n*
$\mathcal{E}$	${\mathbb{N}}^{m}$	A set of edges with size *m*
*G* = ($\mathcal{V}$, $\mathcal{E}$)	${\mathbb{N}}^{n\times m}$	A network defined as an undirected graph with $\mathcal{V}$ and $\mathcal{E}$
$\mathcal{N}(\nu )$	${\mathbb{N}}$	The set of neighbor of vertex $\nu$
$k$	${\mathbb{R}}$	Average node degree in a graph
$d$	${\mathbb{R}}$	Density for an undirected graph
${\tau }_{i}$	[0, 1]	Threshold value of ${\nu }_{i}$ in diffusion model
${S}_{t}^{(i)}$	{0, 1}	State of ${\nu }_{i}$ at the end of week *t* observed from the dataset
${\widehat{S}}_{t}^{(i)}$	{0, 1}	State of ${\nu }_{i}$ at the end of week *t* generated from the model

### First optimization step: obtain parameterized diffusion model

In the first stage optimization, we have 2,010 variables {${\tau }_{1},{\tau }_{2},\dots ,{\tau }_{2010}$} and each ${\tau }_{i}$ could take any real number between 0 and 1. Obviously, solving this problem would be prohibitively expensive. To solve this problem efficiently, we applied a genetic algorithm (GA) to solve the optimization problem. GA is an optimization algorithm used to solve challenging real-world problems encountered in various fields. In this study, we optimized the recovery diffusion model using a classic GA described as described in Algorithm 1. The chromosome representation consists of a series of thresholds {${\tau }_{1},{\tau }_{2},\dots ,{\tau }_{2010}$}. During the initialization of the algorithm ($\theta$ = 0), a population of $\eta$ chromosomes is randomly generated, and their fitness values are calculated. In this study, the fitness function for each chromosome is defined as the reciprocal of loss function $L\left(S,\widehat{S}\right)$. Notably, the population size $\eta$ remains consistent across all generations. In the selection operator, a pair of chromosomes with the highest fitness value is initially chosen. Subsequently, selection operators are performed $\frac{\eta }{2}-1$ times, based on the probability distribution weighted by the fitness value. This process is carried out to generate the next generation while maintaining the population size $\eta$. Additionally, we employ single-point crossover on the selected pair of parent chromosomes to introduce genetic diversity and potentially generate new genetic sequences within the population. During the crossover process, a random crossover point is determined. Two offspring are then created by swapping the tails of the two parent chromosomes beyond the randomly chosen crossover point. After crossover, the generated offspring undergo mutation operation. In GA, a mutation introduces small, random changes in individual chromosomes to explore new solutions. During mutation operation, a position within the chromosome is randomly selected. There is a 50% chance that the selected position will remain the same, while there is a 50% chance that it will be assigned a random number between 0 and 1.

**Figure:**
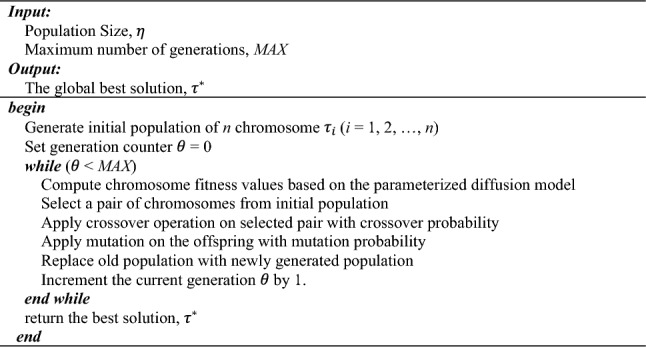
Algorithm 1 Classical Genetic Algorithm (GA)

**Figure 4:**
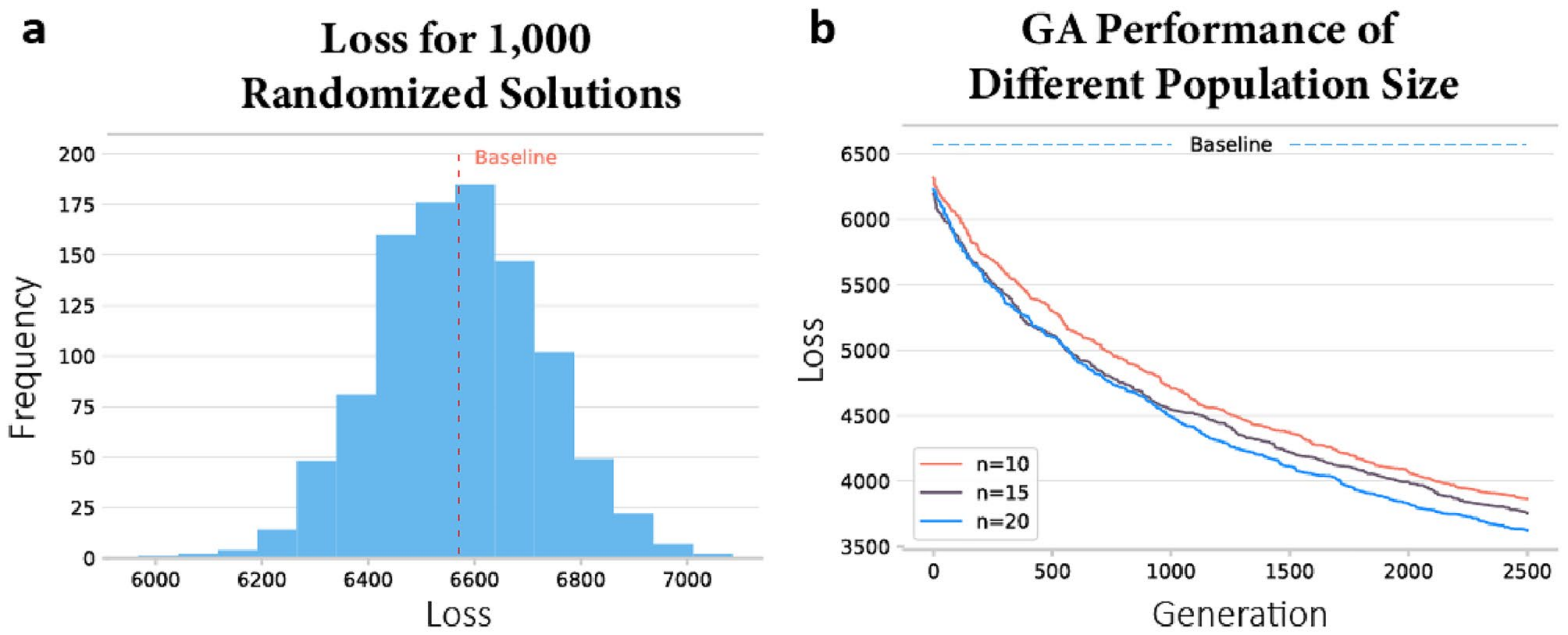
(**a**) The loss of 1,000 randomized recovery diffusion processes and the mean as the baseline. (**b**) The loss of the algorithm with population size $\eta$ equal to 10, 15, and 20.

GA as a population-based metaheuristic algorithm has a proven ability to avoid becoming stuck in the local minimum. The larger the population size $\eta$, the greater the chance that a global optimal solution can be found. However, the programming runtime also increases significantly with increasing $\eta$. To find a promising population size $\eta$ to get a high-quality solution with tolerable runtime, we compared three different population sizes $\eta$ equal to 10, 15, and 20 under the maximum number of 2,500 generations. Due to the stochastic nature of the formation of the initial population, we also performed randomized diffusion processes 1,000 times to get the loss $L\left(S,\widehat{S}\right)$ equal to 6,569.835 to set as the baseline. To compare their performance, we define the algorithm performance ${P}^{(\eta )}$ as

3 $${P}^{(\eta )}=\frac{\Delta {L}^{(\eta )}}{{R}^{(\eta )}}$$

where $\Delta {L}^{(\eta )}$ represents the loss descent rate per generation and ${R}^{(\eta )}$ represents the runtime (in seconds) required for each generation. As shown in Fig. 4 and Table 2, at $\eta$ = 20, the algorithm has the lowest loss with, but also needs the longest runtime among three cases and thus generates the lowest ${P}^{(\eta )}$. On the other hand, the algorithm achieves the best performance index ${P}^{(\eta )}$ with a population size $\eta$ of 10. Therefore, in this study, we will set the population size $\eta$ = 10 and *MAX* = 10,000 to generate the optimal thresholds ${\tau }^{*}=({\tau }_{1}^{*},\dots ,{\tau }_{2010}^{*})$ for 2,010 CBGs in Harris County, Texas.

**Table 2:** The algorithm performances with population size $\eta$ equal to 10, 15, and 20.

Population size $\eta$	Initial loss	Final loss	$\Delta {L}^{(\eta )}$	${R}^{(\eta )}$	${P}^{(\eta )}$
10	6319	3867	0.9808	3.569	0.2748
15	6194	3775	0.9768	6.786	0.1426
20	6227	3624	1.0412	13.021	0.08

### Second optimization step: identify recovery multiplier

After obtaining the parameterized threshold model through GA, we want to identify the critical spatial areas (CBGs), which we call the recovery multipliers, whose recovery would expedite the recovery of the entire community. The idea here is that by selecting a set of CBGs and defining them as *recovered* from the beginning of the diffusion process, the model can finish the recovery process in the shortest period of time. In essence, by strengthening a specific subset of the CBGs in the spatial network topology, we can expedite the recovery of the entire region. In the diffusion model, we set recovery multipliers of size *N* (${\mathcal{M}}^{N}$) as *recovered*, and simulate the network diffusion process by the parameterized model we learned from the first stage optimization. We selected four different sizes of recovery multiplier: *N* equals {20, 60, 101, 201} representing {1%, 3%, 5%, 10%} of the total CBGs. These percentages were selected to represent a range of intervention measures, from relatively small interventions (1%) to larger interventions (10%). By examining the effects of different sized recovery multipliers, we can gain insight into how the recovery of different spatial areas affects the overall community recovery time. This helps in understanding the potential outcomes of different policy or intervention strategies. We set the maximum number of generation *MAX* to 2000 in the second stage of optimization.

## Results

### Empirical threshold parameters

**Figure 5:**
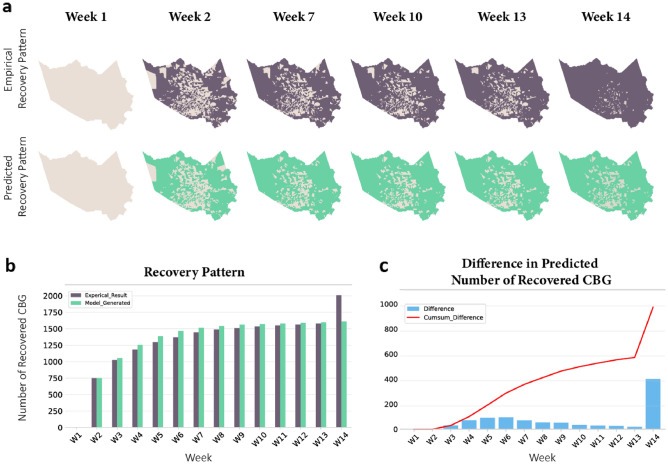
(**a**) The recovery pattern simulated by the network diffusion model on the activity data. (**b**) The number of recovered CBG in each week obtained from the empirical data versus diffusion model. (**c**) The difference and the accumulated difference in recovered CBG between the empirical data and simulated data.

In this section, we present the results of the first- and second-stage optimization. In the first stage, we implement the network diffusion model to specify the empirical threshold values. By applying GA to the population activity recovery (measured by visits to POI) in Harris County after Hurricane Harvey, we obtain the empirical threshold values for each census block group (CBG). Specifically, we set population size $\eta$ = 10 and *MAX* = 10,000 to generate the optimal thresholds ${\tau }^{*}=({\tau }_{1}^{*},\dots ,{\tau }_{2010}^{*})$ for 2,010 CBGs in Harris County, Texas. The optimization algorithm results in a final loss $L\left(\cdot \right)$ of 2752. The results show that the parameterized model is able to capture the spatial effects among communities and then generate the recovery diffusion process through the underlying network structure. Figure 5 shows that the differences in the number of recovered CBGs per week between the empirical data and the simulated results are all below 100, except for the difference at week 14. The reason for the apparent difference (401) at week 14 is due to the research window in processing the population activity recovery duration. To eliminate seasonality effects (effects of holiday season) on population activities, any CBG recovery taking longer than 14 weeks, was designated as 14 weeks (3.5 months). Therefore, any CBG that has not recovered by the end of this window is considered to be recovered by the end of week 14 in the empirical data, which explains the spike in the difference at week 14. The results show that the model has a good ability to simulate and reconstruct the trajectory of the recovery process after the hurricane through the underlying network diffusion process.

### Specifying recovery multipliers

**Figure 6:**
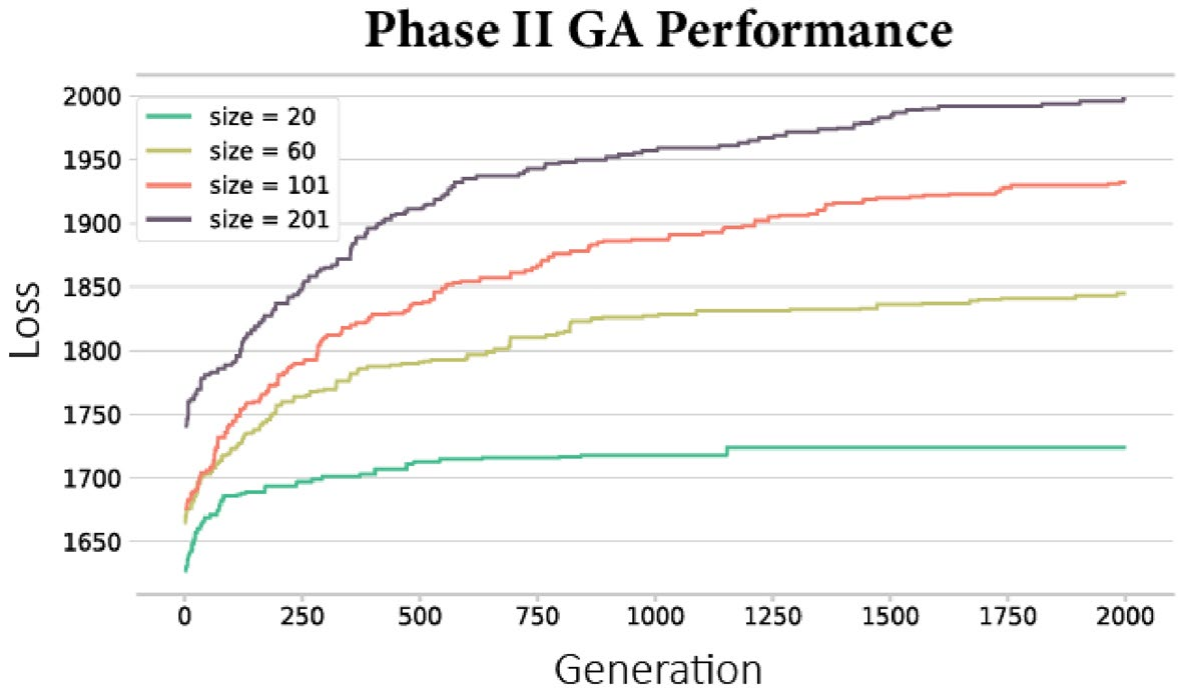
The algorithm performance in terms of the number of recovered CBG at the end of week 14.

**Figure 7:**
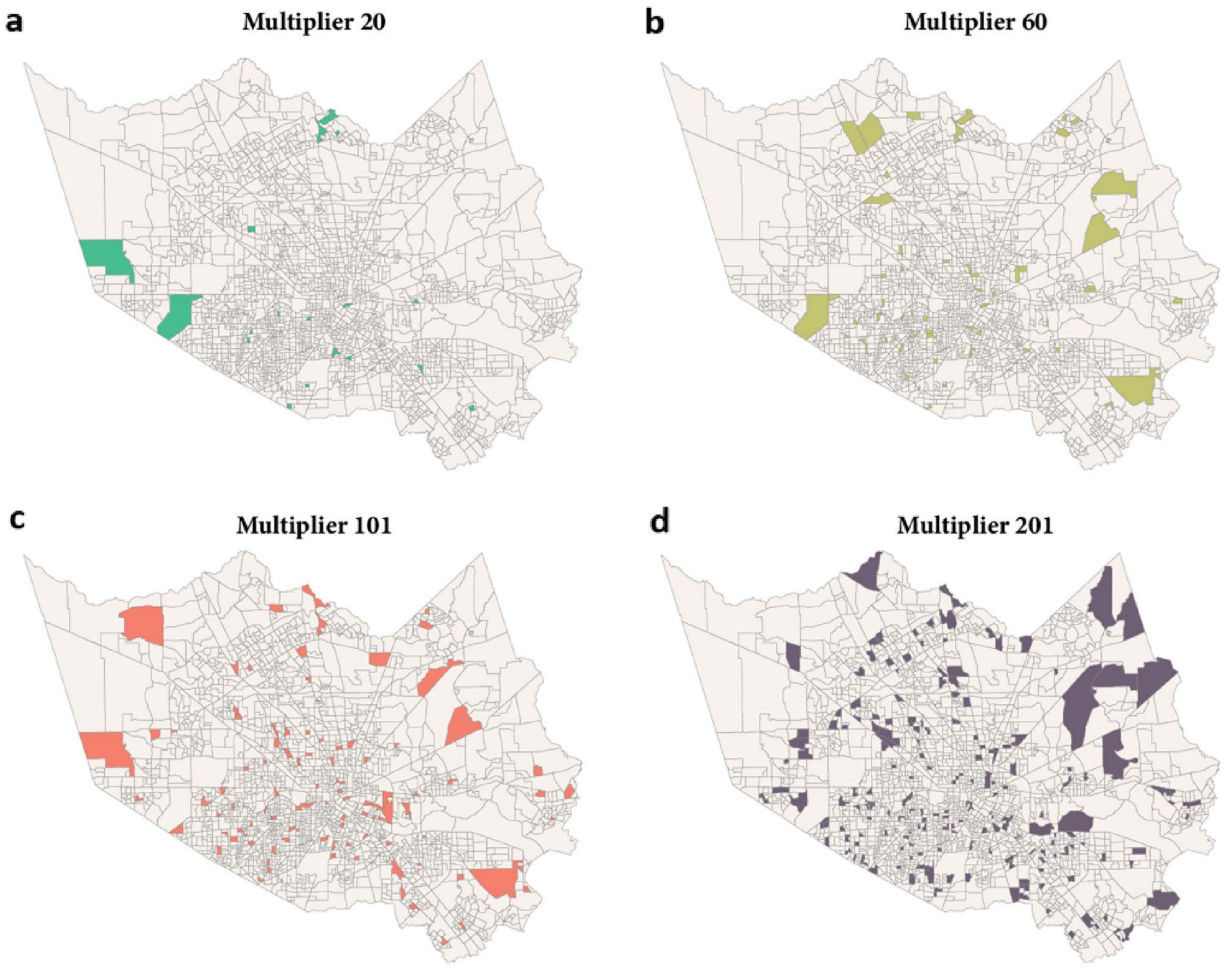
(**a**–**d**) corresponds to the geography of the optimized set ${\mathcal{M}}^{N}$ with *N* equal to 20, 60, 101, and 201.

In the second-phase optimization, we use the parameterized diffusion model to specify the recovery multipliers in the simulated recovery process. A total of 1,609 CBGs were recovered at the end of week 14, leaving 401 CBGs in the *affected* states. We performed the GA to optimize the set of ${\mathcal{M}}^{N}$, which is defined as *recovered* at $\theta$ = 0, in four different cases: *N* $\in$ {20, 60, 101, 201}, which are {1%, 3%, 5%, 10%} of the CBGs throughout the county. The objective function is to maximize the number of recovered CBGs at the end of the diffusion process (*t* = 14) by choosing the right set of ${\mathcal{M}}^{N}$. Figure 6 and Table 3 show that when using the optimized recovery multipliers, the number of recovered CBGs increases by {5.96%, 13.09%, 15.27%, 14.76%} for *N* = {20, 60, 101, 201}, respectively. This represents a significant improvement in the recovery of the entire region. As shown in Fig. 7, we geographically identify the recovery multipliers in the four different cases. These CBGs are the critical recovery spreaders whose fast recovery would increase and expedite the recovery of the entire community.

**Table 3:** The number of recovered CBGs before and after the optimization and the increment rate $(\frac{\#recovered \,with \,optimization-\#recovered \,without \,optimization}{\#recovered \,without \,optimization}\times 100\%)$.

*N*	#Recovered CBG at $\theta$ = 0	#Recovered CBG at $\theta$ = 2000	Increment (%)
20	1627	1724	5.96
60	1665	1845	13.09
101	1676	1932	15.27
201	1741	1998	14.76

### Threshold value analysis

By modeling post-disaster community recovery as a network diffusion process, we characterize the spatial effect of recovery among neighborhoods and spatial areas. In particular, the model shows that the spatial effects are heterogeneous, indicated by varying thresholds in our diffusion model. Essentially, the threshold represents the minimum percentage of recovered neighborhoods before the entire community is considered to be recovered. A higher threshold means more recovered neighbors are located around the CBG before it is recovered. This indicates that a clearer spatial effect was perceived around the CBG. The mean and variance of the threshold $\tau$ are 0.345 and 0.125, respectively. A greater spatial effect implies that there is more spatial interdependence for recovery among areas. Areas with low spatial effect could be resourceful and self-sufficient and thus able to recover with minimal reliance on the resources and facilities of neighboring areas. Such minimal reliance on resources and facilities of neighboring areas would create high spatial interdependence among areas for recovery that would lead to a higher spatial effect in recovery diffusion.

**Figure 8:**
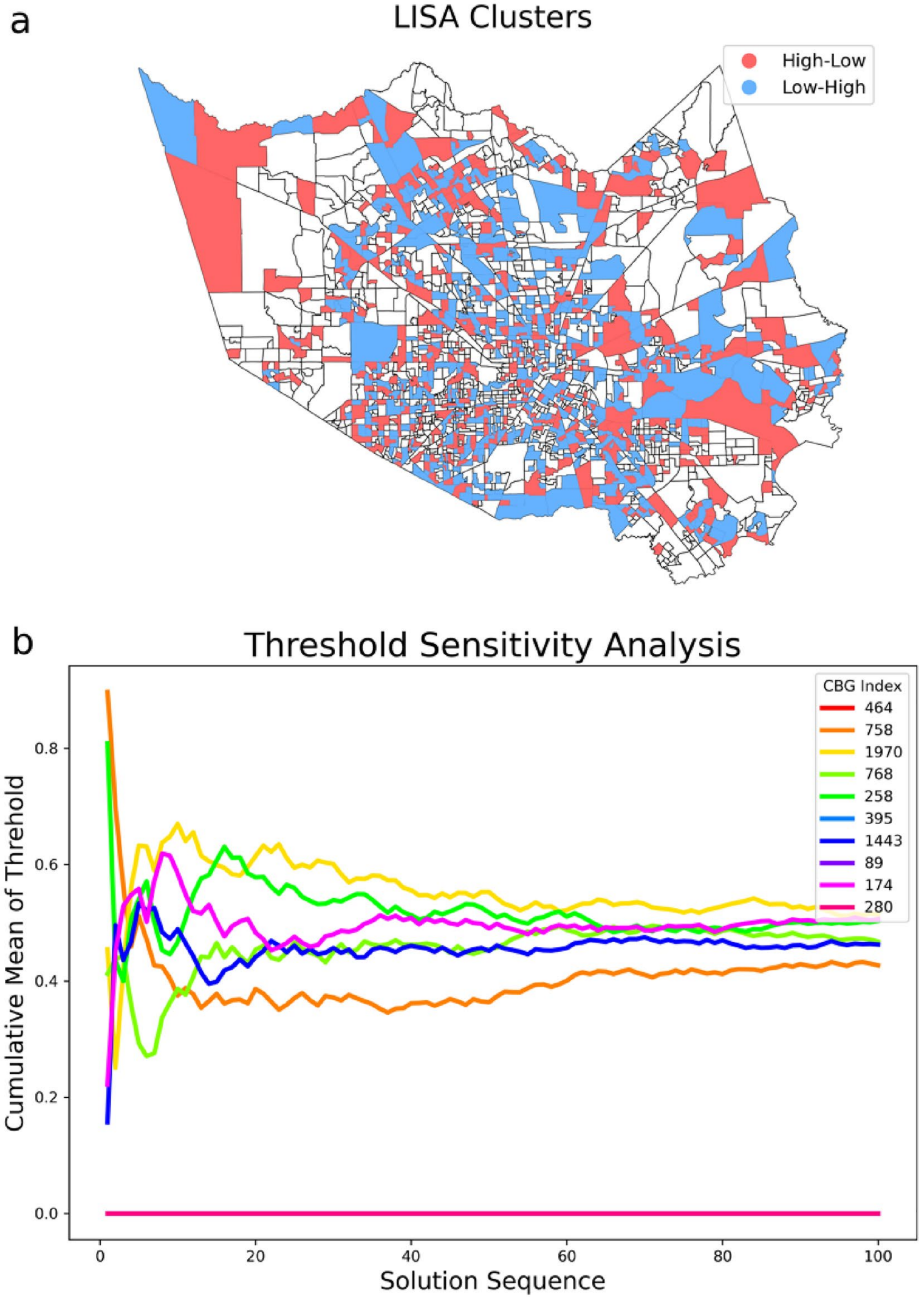
(**a**) The spatial distribution of LISA high–low and low–high categories. (**b**) Cumulative mean of thresholds for a randomly selected set of ten CBGs.

To assess the spatial effects of recovery, we conducted a Local Indicators of Spatial Autocorrelation (LISA) analysis^27^ in the studied area. LISA is a spatial analysis technique that focuses on identifying local patterns to assess clustering within a geographic region. In our analysis, we specifically examined two of four possible groups: (1) CBGs are categorized as High-Low (HL) if they possess high threshold values but are surrounded by neighboring areas with low thresholds. Addressing these areas can potentially facilitate the recovery of the surrounding areas with lower thresholds. (2) CBGs fall into the Low–High (LH) category if they have low thresholds but are surrounded by neighboring areas with high thresholds. This suggests that these areas could be easily recovered if the surrounding area were recovered. Figure 8a shows the spatial distribution of LISA clusters. To evaluate the robustness of the optimal threshold, we performed a sensitivity analysis by running the diffusion model 100 times. Figure 8b shows the cumulative average of the thresholds for a randomly selected set of 10 CBGs and shows convergence as the size of the solution sequence increases.

### Recovery multiplier analysis

**Figure 9:**
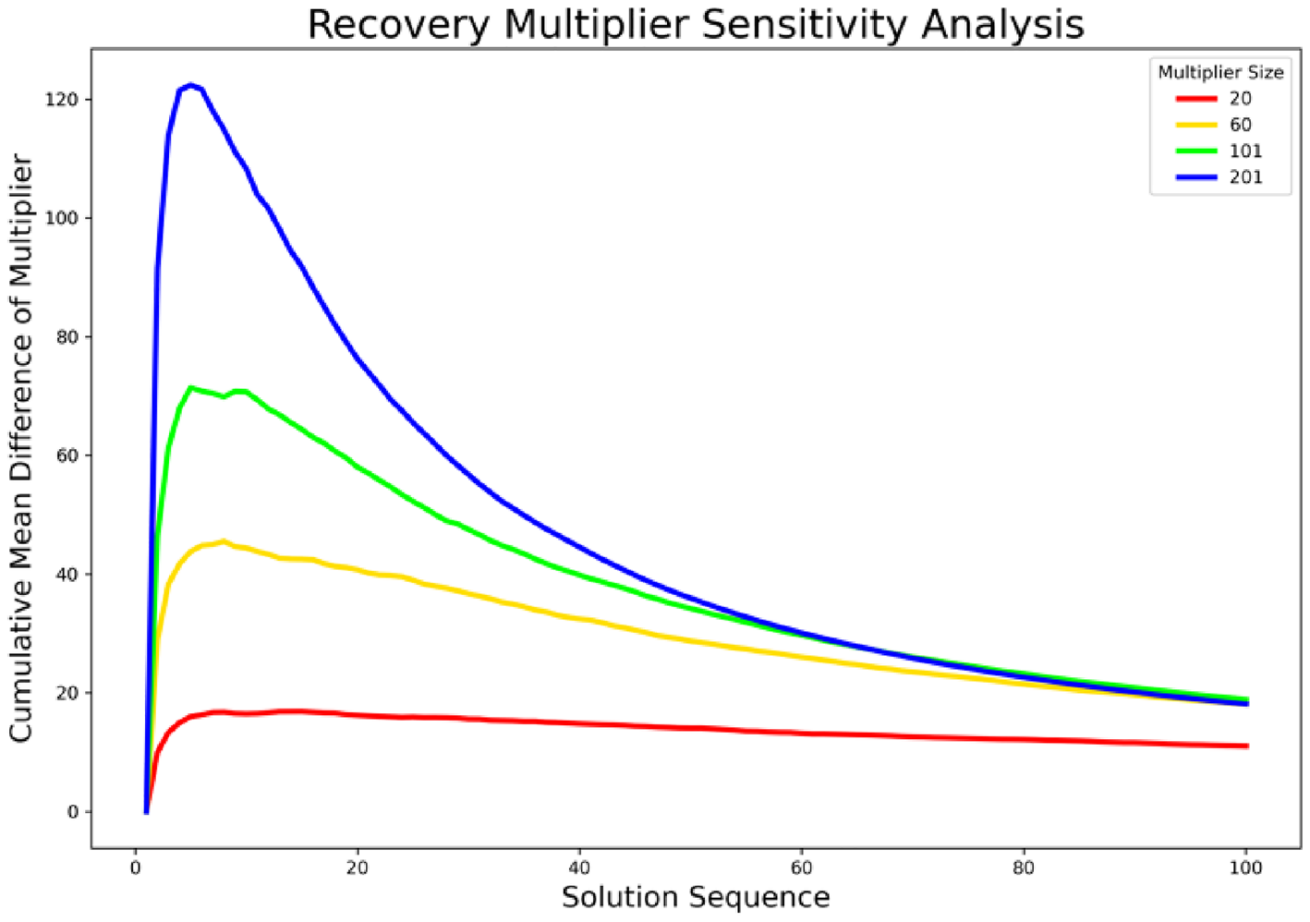
Cumulative mean difference in optimal multiplier set.

**Figure 10:**
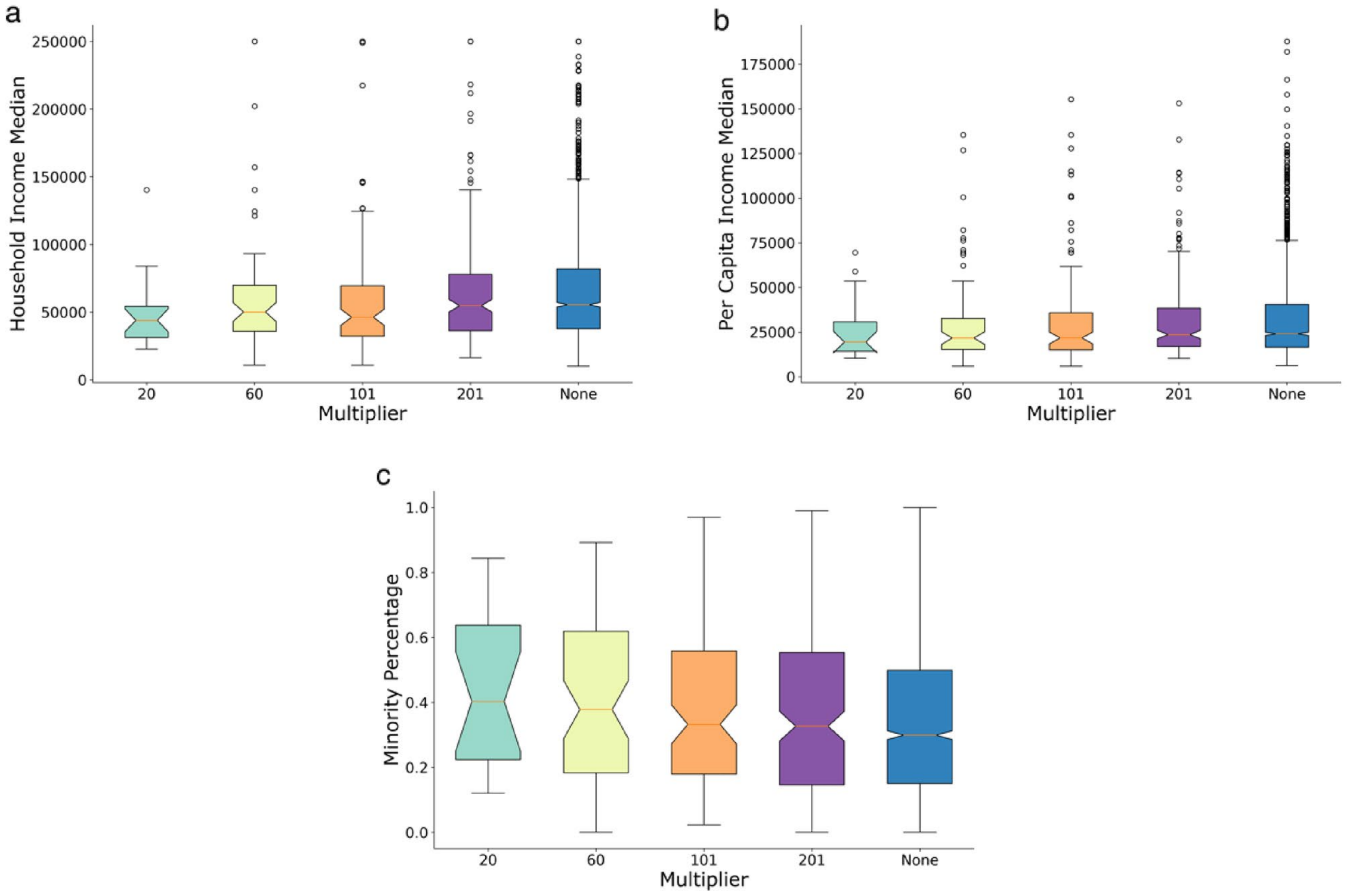
(**a**) The household median distribution of the recovery multiplier with different sizes. (**b**) The per capita income median distribution of the recovery multiplier with different sizes. (**c**) The minority percentage distribution of the recovery multiplier with different sizes.

In this section, we conduct a sensitivity analysis of the recovery multiplier. To assess the robustness of the optimal multipliers, we run the second-stage optimization model 100 times to derive different sets of optimal recovery multipliers. Figure 9 shows that the cumulative mean difference of the multipliers in four different cases consistently converges to values below 20 as the solution sequence increases. In the next step, we examine the recovery multipliers in terms of their social-demographic attributes. Previous studies have highlighted the heterogeneity of recovery in disaster-affected neighborhoods and the influential role of socio-demographics in shaping a community’s recovery trajectory^28–30^. To understand social disparities in recovery following the studied hazard event, we conducted a spatial effects analysis with respect to the socio-demographic attributes of CBGs. We used three variables, median household income, per capita income, and minority percentage both measured at the block group level by the U.S. Census Bureau. Two notable findings were observed (Fig. 10) from the analysis: (1) With a smaller number of multipliers selected, the identified multipliers are areas with lower per capita/household income; also in all four cases of multiplier sizes, multipliers have lower per capita/household income than the non-multiplier; and (2) In the case of smaller multiplier size, the multipliers have a larger minority percentage, and in all four cases, multipliers have a higher minority percentage than the non-multiplier. The two results imply that identifying the recovery multipliers and allocating resources to them not only improve the equality aspects of recovery for the vulnerable population but also play an important role in the diffusion of recovery in the entire community. These multipliers are the neighborhoods that need to be prioritized for resource allocation in the recovery period to improve both the equity and speed of recovery in the entire community. This result is significant since it shows improving equity in recovery resource allocation could benefit the recovery of the entire community.

## Discussion

Post-disaster recovery is one of the critical processes of community resilience to hazards. Despite several years of research on the determinants of community recovery, little knowledge exists regarding the spatial network processes influencing community networks. Socio-spatial networks are structures upon which various processes related to community resilience unfold. In this study, we investigated community recovery as a network diffusion process in order to evaluate the extent of spatial contiguity effects in the spread of recovery across different areas of community and also identify ways to expedite the recovery of the entire community through activating network effects with the specification of recovery multipliers.

In this study, we chose to use the queen-based neighborhood network for two reasons. First, queen contiguity takes into account both the presence of a common edge and a common vertex between two spatial units. This inclusivity allows us to capture various types of spatial connections, which is crucial in the context of disaster recovery where interactions can occur through multiple channels. While it is true in many urban and social studies that spatial interactions can be hierarchical, disaster recovery often involves multiple factors and mechanisms. In our case, we aimed to understand recovery diffusion comprehensively, taking into account both strong and weak connections between neighboring areas. Second, the choice of queen neighborhood helps to more clearly characterize spatial diffusion during the post-disaster period. This is particularly relevant as livelihoods often depend on local businesses at this time due to the limited number of access routes and the desirability of minimizing long-distance travel. However, we recognize that in other contexts or for certain research questions, other network structures such as hierarchical or weighted networks may be more appropriate. Our choice of queen contiguity was based on the need to explore the full spectrum of spatial interactions in the context of post-disaster recovery.

The findings of this study provide important scientific and practical contributions. This study is the first research to investigate network effects in post-disaster community recovery. While prior studies had identified the presence of disparities in the recovery speed of areas, the majority of studies attribute the slow recovery to sociodemographic attributes. The findings of this study reveal the heterogeneity of spatial contiguity effects in community recovery. Furthermore, prior studies have reported the presence of slow recovery hotspots in the communities; however, limited knowledge exists about mechanisms that contribute to the clustering of slow recovery areas. Our findings resolve this unknown by showing that a CBG surrounded by other socially vulnerable areas and the absence of the required spatial effect for the diffusion of recovery could create recovery isolates (hotspot clusters of CBGs from socially vulnerable groups that struggle to recover due to the absence of spatial effect from the neighboring areas). In addition, the findings of this study introduce the novel concept of recovery multipliers and show that focusing on socially vulnerable areas as recovery multipliers not only enhances the equity of post-disaster recovery but also expedites the recovery for the entire community. From a practical perspective, the model in this study could be used to proactively monitor community recovery and predict the diffusion of recovery. Also, the findings of this study have important implications for public officials, emergency managers, and decision makers involved in disaster recovery to better leverage the diffusion processes to accelerate and also promote equity in post-disaster community recovery. For example, identifying and prioritizing recovery multipliers for recovery resource allocation greatly enhance the speed of recovery in the entire community and also augment recovery equity.

As the first study focusing on network dynamics of community recovery, the findings of this study open avenues for subsequent areas of inquiry for future studies. For example, future studies can examine various social characteristics, such as household composition, ethnic minority status, and housing conditions, and consider a range of built environment factors, including transport access and infrastructure service availability. These investigations may elucidate a possible connection with the spatial contiguity effect in community recovery diffusion. Furthermore, there is ample scope for future research to expand its horizons by examining spatial effects and recovery diffusion across different hazard events and regions. This more comprehensive analysis would enable the identification of general characteristics inherent in community recovery diffusion within spatial networks. Addressing the lines of inquiry focusing on recovery network dynamics would bring us closer to better understanding and improving post-disaster recovery of communities through the lens of network processes.

## Data Availability

All data were collected through a CCPA- and GDPR-compliant framework and utilized for research purposes. No human subjects are recruited in the research process. The data that support the findings of this study are available from Spectus, but restrictions apply to the availability of these data, which were used under license for the current study. The data can be accessed upon request submitted on Spectus (https://spectus.ai/). Other data we use in this study are all publicly available.

## Code availability

The code that supports the findings of this study is available from the corresponding author upon request.

## References

[1] Burton CG (2015). A validation of metrics for community resilience to natural hazards and disasters using the recovery from Hurricane Katrina as a case study. Ann. Assoc. Am. Geogr..

[2] Davidson, R. & Cagnan, Z. Restoration Modeling of Lifeline Systems. (2004).

[3] Miles SB, Chang SE (2003). Urban Disaster Recovery: A Framework and Simulation Model.

[4] Stevenson JR, Emrich CT, Mitchell JT, Cutter SL (2010). Using building permits to monitor disaster recovery: A spatio-temporal case study of Coastal Mississippi following Hurricane Katrina. Cartogr. Geogr. Inf. Sci..

[5] Cohen O, Leykin D, Lahad M, Goldberg A, Aharonson-Daniel L (2013). The conjoint community resiliency assessment measure as a baseline for profiling and predicting community resilience for emergencies. Technol. Forecast. Soc. Change.

[6] Hikichi H (2020). Community-level social capital and cognitive decline after a natural disaster: A natural experiment from the 2011 Great East Japan Earthquake and Tsunami. Soc. Sci. Med..

[7] Meyer MA, Rodríguez H, Donner W, Trainor JE (2018). Social capital in disaster research. Handbook of Disaster Research.

[8] Norris FH, Stevens SP, Pfefferbaum B, Wyche KF, Pfefferbaum RL (2008). Community resilience as a metaphor, theory, set of capacities, and strategy for disaster readiness. Am. J. Community Psychol..

[9] Sherrieb K, Norris FH, Galea S (2010). Measuring capacities for community resilience. Soc. Indic. Res..

[10] Dash N, Morrow BH, Mainster J, Cunningham L (2007). Lasting effects of Hurricane Andrew on a working-class community. Nat. Hazards Rev..

[11] Zottarelli LK (2008). Post-Hurricane Katrina employment recovery: The interaction of race and place*. Soc. Sci. Q..

[12] Liu, C.-F. & Mostafavi, A. *Hazard Exposure Heterophily: A Latent Characteristic in Socio-Spatial Networks Influencing Community Resilience*. http://arxiv.org/abs/2205.01868 (2022). 10.48550/arXiv.2205.01868. 10.1038/s41598-023-31702-9 PMC10039027 36964245

[13] Lee, C.-C., Namburi, S., Xiao, X. & Mostafavi, A. *Homophilic and Heterophilic Characteristics Shaping Community Formation in Human Mobility Networks During Extreme Weather Response*. http://arxiv.org/abs/2205.04981 (2022). 10.48550/arXiv.2205.04981.

[14] Pezzica C, Chioni C, Cutini V, Bleil de Souza C, Gervasi O (2020). Assessing the impact of temporary housing sites on urban socio-spatial performance: The case of the Central Italy earthquake. Computational Science and Its Applications—ICCSA 2020, pp 324–339.

[15] Hillier B, Hanson J (1989). The Social Logic of Space.

[16] Coleman, N., Liu, C., Zhao, Y. & Mostafavi, A. Lifestyle pattern analysis unveils recovery trajectories of communities impacted by disasters. Preprint at http://arxiv.org/abs/2207.03589 (2022).

[17] Cowan R, Jonard N (2004). Network structure and the diffusion of knowledge. J. Econ. Dyn. Control.

[18] Szor P (2005). The Art of Computer Virus Research and Defense: ART COMP VIRUS RES DEFENSE _p1.

[19] Wang P, González MC, Menezes R, Barabási A-L (2013). Understanding the spread of malicious mobile-phone programs and their damage potential. Int. J. Inf. Secur..

[20] Burt RS (1987). Social contagion and innovation: Cohesion versus structural equivalence. Am. J. Sociol..

[21] Valente TW (1996). Social network thresholds in the diffusion of innovations. Soc. Netw..

[22] Kermack WO, McKendrick AG, Walker GT (1927). A contribution to the mathematical theory of epidemics. Proc. R. Soc. Lond. Ser. Contain. Pap. Math. Phys. Charac..

[23] Granovetter M (1978). Threshold models of collective behavior. Am. J. Sociol..

[24] Kempe, D., Kleinberg, J. & Tardos, É. Maximizing the spread of influence through a social network. in *Proceedings of the ninth ACM SIGKDD international conference on Knowledge discovery and data mining* 137–146 (Association for Computing Machinery, 2003). 10.1145/956750.956769.

[25] Podesta C, Coleman N, Esmalian A, Yuan F, Mostafavi A (2021). Quantifying community resilience based on fluctuations in visits to points-of-interest derived from digital trace data. J. R. Soc. Interface.

[26] Rossetti G (2018). NDlib: A python library to model and analyze diffusion processes over complex networks. Int. J. Data Sci. Anal..

[27] Anselin L (1995). Local indicators of spatial association—LISA. Geogr. Anal..

[28] Chang SE (2016). Socioeconomic impacts of infrastructure disruptions. Oxf. Res. Encycl. Nat. Hazard Sci..

[29] Cutter SL (2006). The long road home: Race, class, and recovery from Hurricane Katrina. Environ. Sci. Policy Sustain. Dev..

[30] Mcdonnell S (1995). Evaluation of long-term community recovery from Hurricane Andrew: Sources of assistance received by population sub-groups. Disasters.

